# Dietary antioxidant intake in women with facial melasma: a case-control study^؆^

**DOI:** 10.1016/j.abd.2025.501218

**Published:** 2025-10-27

**Authors:** Ana Cláudia Cavalcante Espósito, Carla Rakoski Ribas, Gracielli Ferreira Barbosa, Julia Dias Bendini, Rita Gisele Biffi Medeiros, Carolina Nunhez da Silva, Tatiana Cristina Figueira, Hélio Amante Miot

**Affiliations:** a Department of Medicine, Universidade do Oeste Paulista, Presidente Prudente, SP, Brazil; b Department of Infectology, Dermatology, Imaging Diagnosis and Radiotherapy, Faculty of Medicine, Universidade Estadual Paulista, Botucatu, SP, Brazil

*Dear Editor,*

Melasma is a chronic, multifactorial pigmentary dermatosis that exclusively affects sun-exposed areas (with lesions predominantly on the face) and is more prevalent in women in the period between menarche and menopause. Genetic predisposition, exposure to ultraviolet radiation (UVR), and hormonal stimuli are its main triggers. However, other factors, such as exposure to environmental pollution, heat, sleep deprivation, and chronic conditions such as depression, can aggravate systemic oxidative stress, potentially contributing to the genesis and progression of melasma.^1^

Systemic oxidative stress is prominent in patients with melasma. Concurrently, the administration of oral antioxidants, such as pycnogenol, enhances the clearing effect of topical treatment.^2^

Chronic exposure to UVR, especially type A, induces both cell and systemic oxidative stress, contributing to the sustained melanogenesis observed in melasma.^1^ There is a negative correlation between the clinical severity of melasma and plasma glutathione (GSH) levels, as well as increased levels of components of the intracellular antioxidant defense enzyme system, such as superoxide dismutase (SOD) and GSH-peroxidase.^3^ Furthermore, individuals with melasma have higher plasma concentrations of malondialdehyde, a byproduct of lipid peroxidation that is a marker of cell damage.^4^

In the context of antioxidant defense mechanisms, in addition to the enzyme system (such as SOD and GSH-peroxidase), the importance of non-enzymatic antioxidants obtained through diet stands out. These include compounds present in foods rich in beta-carotene, vitamins C and E, ellagic acid, bioflavonoids, catechins, isoflavones, lycopene, omega-3 fatty acids, polyphenols, resveratrol, selenium, zinc, and cysteine.

To date, no study has systematically evaluated dietary aspects in individuals with melasma. To fill this gap, a cross-sectional study was conducted to investigate dietary patterns, with a specific focus on the intake of antioxidant-rich foods, in women with facial melasma compared to those without the condition.

After approval by the institutional ethics committee (CAAE 26390419.7.0000.5515), 194 adult women of childbearing age (aged 18 to 49) were included and divided into two groups: 97 with facial melasma (diagnosed by a dermatologist) and 97 controls. Participant recruitment occurred from September 2023 to January 2024. Matching was performed by age, and women with concomitant facial dermatoses were excluded. Sampling was consecutive, among patients treated at the Presidente Prudente Regional Hospital, and was conducted after informed consent was obtained.

The main study outcome measure was the adapted Food Frequency Questionnaire (FFQ), which was administered to participants to assess their weekly consumption of 41 types of foods (Supplementary Material). The data obtained were converted to grams, and from this, energy, macronutrient, and micronutrient values were calculated using the Nutritional Data System for Research (NDSR) software. Dietary patterns were analyzed based on their components, extracted using the PLS (Partial Least Squares) method, using data scaled by standard deviations.^5^ All dietary intake assessments were processed and analyzed by a nutritionist (TCF).

In addition, demographic data, adherence to photoprotection, smoking status, and oral antioxidant supplementation were collected. Nutrients were compared between groups using the Mann-Whitney (exact) test, with significance set at p ≤ 0.01. Sample size was calculated to detect a minimum difference of 20% in nutrient intake between groups, with an alpha of 0.01 and a beta of 0.1.

There were no differences between the groups in age, phototypes, age at menarche, smoking status, number of children, adherence to sunscreen, contraceptive use, or oral antioxidant supplementation (Table 1).

**Table 1:** Main demographic data of women with melasma and controls.

Variables	Melasma	Control	p-value^a^
*n*	97	97	‒
Age (years) – mean (sd)	38 (7)	37 (9)	0.162
Phototype (Fitzpatrick) – n (%)			0.035
I‒II	37 (38)	50 (52)	
III	44 (45)	39 (40)	
IV‒V	16 (17)	8 (8)	
Family member with melasma – n (%)	58 (60)	20 (21)	**<0.001**
Regular use of sunscreen – n (%)	73 (75)	63 (65)	0.158
Age at menarche (years) – mean (sd)	12 (1)	12 (1)	0.916
Time of melasma (years) – median (p25–p75)	8 (5–15)	‒	‒
Number of children – median (p25–p75)	1 (0–2)	1 (0–2)	0.243
Uses oral contraceptive– n (%)	33 (34)	39 (40)	0.458
Current smoker– n (%)	9 (9)	2 (2)	0.058
Use of oral antioxidant– n (%)	18 (19)	10 (10)	0.152

a Bivariate analysis.

The diet in both groups was monotonous, and the most commonly consumed foods per week in both groups were milk, cheese, oranges, tomatoes, coffee, and white meat. No celiac disease or vegetarians were found in the sample. Extraction of three PLS components based on the dietary patterns formed by the 41 foods studied explained the variance of only 20% of the groups, without implying adequate separation between the melasma group and controls (Fig. 1). Furthermore, no differences were observed between the two groups regarding the consumption of macro and micronutrients, including dietary antioxidants (Table 2).

**Fig. 1:**
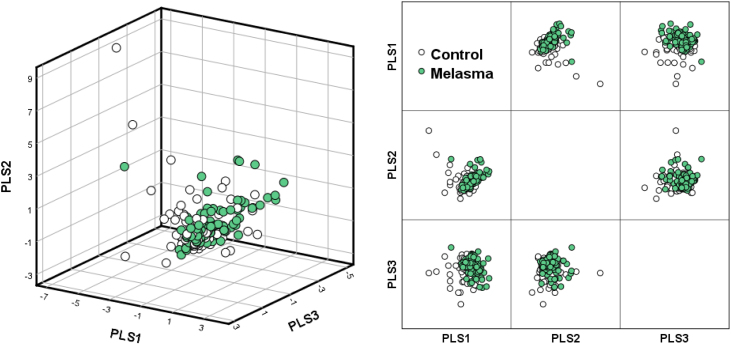
Dietary patterns of participants regarding the 41 foods investigated. Loading according to the three PLS (parcial least squares) components (n = 194).

**Table 2:** Nutritional data of participants' diets, reported in one week (n = 194).

Nutrients	Melasma			Control			p-value^a^
	Median	p25	p75	Median	p25	p75	
Energy(kcal)	18,164	12,396	25,836	21,110	13,309	29,083	0.208
Protein (g)	920	635	1,418	1,204	715	1,878	0.023
Carbohydrate (g)	2,392	1,292	3,618	2,332	1,365	3,224	0.608
Lipids (g)	441	328	666	598	335	952	0.021
Saturated lipids (g)	163	71	311	223	86	407	0.053
Monounsaturated Lipids (g)	116	61	168	173	81	282	0.024
Polyunsaturated Lipids (g)	53	32	100	61	33	124	0.258
Cholesterol (mg)	2,427	1,564	4,815	3,240	1,799	7,157	0.063
Fiber (g)	438	235	745	384	251	735	0.924
Sodium (mg)	13,817	7,736	30,341	19,383	11,864	33,522	0.039
Calcium (mg)	13,744	7,575	20,338	14,368	8,832	23,694	0.205
Iron (mg)	107	63	207	133	75	217	0.213
Zinc (mg)	95	63	140	124	70	156	0.055
Vitamin A (mcg)	11,617	5,259	23,800	10,542	4,818	24,518	0.967
Vit. B1 (mg)	20	11	28	21	13	34	0.273
Vit. C (mg)	4,586	1,895	9,793	3,678	1,523	7,917	0.220
Vitamin E (mg)	82	45	166	105	45	182	0.467
Vitamin B6 (mg)	29	20	46	34	21	44	0.419
Vitamin D (mg)	23	9	65	34	9	98	0.103
Potassium (mg)	52,573	32,826	77,674	55,828	35,776	78,289	0.675
Magnesium (mg)	4,774	2,987	7,318	5,491	3,142	7,981	0.257
Selenium (mg)	878	508	1,570	1,044	568	2,234	0.086
Folate (mg)	4,388	2,099	6,665	3,899	2,323	6,163	0.627

a Bivariate analysis.

Despite the oxidative microenvironment in the upper dermis, the causes of systemic oxidative imbalance in melasma patients are not understood, and the results of this study demonstrated no differences in dietary antioxidant consumption in these patients.

The eating habits of the urban Brazilian population are unbalanced, characterized by low food variability, high caloric intake of carbohydrates such as rice and pasta, and consumption of ultraprocessed foods, but low intake of fiber and micronutrients.^6^ The study participants did not exhibit eating habits that differed from those of the urban Brazilian population.

Research on dietary patterns and their association with health outcomes is important but has been little explored systematically in the dermatological literature. It is also important to highlight that understanding the nutritional components of a diet influences not only the body's nutritional aspects but also the composition of the intestinal microbiome, the effects of xenobiotics, and the overall epigenetic control exerted by certain foods.^7^

There are no robust studies in the literature that have investigated the increased consumption of antioxidant-rich foods in other dermatoses. Specifically in rosacea, studies with zinc supplementation have shown conflicting results and methodological limitations, resulting in insufficient evidence to recommend its clinical use. A review of the literature on dietary supplements showed that, in patients with vitiligo, only oral vitamin D replacement is recommended in cases of proven deficiency, although there is still no consensus on the ideal dose to promote repigmentation. The use of *Polypodium leucotomos* is also indicated, as long as it is combined with phototherapy, and shows evidence of increased repigmentation, especially in exposed areas. Supplementation with vitamins C and B12, vitamin E, antioxidant mixtures, and *Ginkgo biloba* still has insufficient evidence for routine recommendation.^8^

A Chinese study involving 150 cases and 150 controls identified alcohol intake as a risk factor (Odds Ratio = 20.5) and soft drink consumption as a protective factor (Odds Ratio = 0.04) for the development of melasma. However, these factors have not been adequately investigated from a pathophysiological perspective.^9^ And, despite being a common demand of patients, there are still no validated associations between dietary aspects and the onset of melasma. In this study, no differences in dietary patterns were identified when comparing women with melasma and the control group.

To date, evidence suggests oral supplementation with antioxidants (such as pycnogenol) as an adjunct to treatment.^10^ In the context of specific dietary intake, it is hypothesized that soy may exacerbate melasma due to its "estrogen-like" action; however, there is no data on a critical amount for this outcome.^11^

Studies related to dietary patterns have limitations related to participant recall, food recall, and the monotony of urban populations' diets. However, sample homogeneity, such as that of these participants from a single center, strengthens internal validity when exploring factors linked solely to diet. The lack of observational association does not mean that dietary interventions are ineffective; however, different study designs are required to confirm this. Finally, studies exploring the intestinal microbiome may suggest dietary interventions for melasma.

In conclusion, neither dietary patterns nor dietary antioxidant consumption is associated with the development of melasma in Brazilian women.

## Authors' contributions

Ana Cláudia Cavalcante Espósito: Design and planning of the study; analysis and interpretation of data; drafting and editing of the manuscript; critical review of the literature; critical review of the manuscript; approval of the final version of the manuscript.

Carla Rakoski Ribas: Design and planning of the study; analysis and interpretation of data; drafting and editing of the manuscript; critical review of the literature; critical review of the manuscript; approval of the final version of the manuscript.

Gracielli Ferreira Barbosa: Design and planning of the study; analysis and interpretation of data; drafting and editing of the manuscript; critical review of the literature; critical review of the manuscript; approval of the final version of the manuscript.

Julia Dias Bendini: Design and planning of the study; analysis and interpretation of data; drafting and editing of the manuscript; critical review of the literature; critical review of the manuscript; approval of the final version of the manuscript.

Rita Gisele Biffi Medeiros: Design and planning of the study; analysis and interpretation of data; drafting and editing of the manuscript; critical review of the literature; critical review of the manuscript; approval of the final version of the manuscript.

Carolina Nunhez da Silva: Design and planning of the study; analysis and interpretation of data; drafting and editing of the manuscript; critical review of the literature; critical review of the manuscript; approval of the final version of the manuscript.

Tatiana Cristina Figueira: Analysis and interpretation of data; drafting and editing of the manuscript; collection of data; approval of the final version of the manuscript.

Hélio Amante Miot: Design and planning of the study; analysis and interpretation of data; statistical analysis; drafting and editing of the manuscript; critical review of the literature; critical review of the manuscript; approval of the final version of the manuscript.

## Approval by the Ethics Committee

This project was approved by the Ethics Committee of Universidade do Oeste Paulista CAAE: 26390419.7.0000.5515 (UNOESTE).

## Financial support

None declared.

## Availability of research data

The entire dataset supporting the results of this study was published in this article.

## Conflicts of interest

None declared.

## References

[1] Espósito A.C.C., Cassiano D.P., da Silva C.N., Lima P.B., Dias J.A.F., Hassun K. (2022). Update on melasma-part I: pathogenesis. Dermatol Ther.

[2] Lima P.B., Dias J.A.F., Esposito A.C.C., Miot L.D.B., Miot H.A. (2021). French maritime pine bark extract (pycnogenol) in association with triple combination cream for the treatment of facial melasma in women: a double-blind, randomized, placebo-controlled trial. J Eur Acad Dermatol Venereol.

[3] Wiraguna A.A.G.P., Hari E.D., Praharsini I.G.A.A. (2020). Correlation between glutathione plasma with degree severity of melasma in balinese women. Clin Cosmet Investig Dermatol.

[4] Choubey V., Sarkar R., Garg V., Kaushik S., Ghunawat S., Sonthalia S. (2017). Role of oxidative stress in melasma: a prospective study on serum and blood markers of oxidative stress in melasma patients. Int J Dermatol.

[5] Yang T.C., Aucott L.S., Duthie G.G., Macdonald H.M. (2017). An application of partial least squares for identifying dietary patterns in bone health. Arch Osteoporos.

[6] Mendonça R.D., Lopes M.S., Freitas P.P., Campos S.F., de Menezes M.C., Lopes A.C.S. (2019). Monotony in the consumption of fruits and vegetables and food environment characteristics. Rev Saude Publica.

[7] Lindell A.E., Zimmermann-Kogadeeva M., Patil K.R. (2022). Multimodal interactions of drugs, natural compounds and pollutants with the gut microbiota. Nat Rev Microbiol.

[8] Jamgochian M., Alamgir M., Rao B. (2023). Diet in dermatology: review of diet’s influence on the conditions of rosacea, hidradenitis suppurativa, herpes labialis, and vitiligo. Am J Lifestyle Med.

[9] Shi Y., Guo S., Tan C. (2025). Diet and living environment as novel etiological factors for melasma: the results from a retrospective case-control study of 150 Chinese patients. J Cosmet Dermatol.

[10] Cassiano D.P., Espósito A.C.C., da Silva C.N., Lima P.B., Dias J.A.F., Hassun K. (2022). Update on Melasma—Part II: Treatment. Dermatol Ther (Heidelb).

[11] Vora R., Khushboo M., Shah A., Patel D., Patel T. (2020). Diet in dermatology: a review. Egypt J Dermatol Venerol.

