# Supplementary material for: Dietary antioxidant intake in women with facial melasma: a case-control study
Source: An Bras Dermatol. 2025 Oct 27;100(6):501218. doi: 10.1016/j.abd.2025.501218 (PMC12596598; doi:10.1016/j.abd.2025.501218)
Supplement: Supplementary file 1 [file mmc1.docx]

**ABD-D-25-00147**

**Supplementary Material**

| **How many times a week and in what quantity do you eat:** | | |
| --- | --- | --- |
| **Food** | **Number of times a week** | **Quantity** |
| Chicken liver |  |  |
| Beef liver |  |  |
| Whole milk |  |  |
| Cheese |  |  |
| Acerola |  |  |
| Broccoli |  |  |
| Cashew |  |  |
| Kale |  |  |
| Spinach |  |  |
| Kiwi |  |  |
| Orange |  |  |
| Lemon |  |  |
| Mango |  |  |
| Melon |  |  |
| Strawberry |  |  |
| Papaya |  |  |
| Tomato |  |  |
| Grapes |  |  |
| Chestnuts |  |  |
| Oatmeal |  |  |
| Prunes |  |  |
| Garlic |  |  |
| Sweet potato |  |  |
| Salmon |  |  |
| Apple |  |  |
| Blackberry |  |  |
| Nuts |  |  |
| Coffee |  |  |
| Peanuts |  |  |
| Brown rice |  |  |
| Guava |  |  |
| Jaboticaba |  |  |
| Açai |  |  |
| Onion |  |  |
| Tuna |  |  |
| Milk |  |  |
| Corn |  |  |
| Egg yolk |  |  |
| Sardine |  |  |
| White meat |  |  |
| Peas |  |  |
